# Medial hinge fracture after closing wedge high tibial osteotomy: Proposing a new classification and risk factor analysis of a neglected complication

**DOI:** 10.1002/ksa.70224

**Published:** 2025-12-08

**Authors:** Gian Andrea Lucidi, Giovanni Balboni, Luca Solaro, Margherita Bonaiuti, Stefano di Paolo, Stefano Zaffagnini

**Affiliations:** ^1^ 2nd Orthopaedic and Trauma Department IRCCS Rizzoli Orthopaedic Institute Bologna Italy; ^2^ Department of Biomedical and Neuromotor Sciences (DIBINEM) University of Bologna Bologna Italy; ^3^ University of Bologna Bologna Italy

**Keywords:** complication, high tibial osteotomy (HTO), hinge fracture, lateral closing wedge

## Abstract

**Purpose:**

Contralateral hinge fracture is one of the most common complications of medial opening wedge high tibial osteotomy (HTO), however, this complication has been poorly investigated after closing‐wedge HTO (CW‐HTO). The primary aim of this study was to describe the incidence and characteristics and propose a classification system of medial hinge fracture (MHF) after CW‐HTO. The secondary aim was to identify demographic and surgical factors that predispose to MHF.

**Methods:**

Consecutive patients who underwent CW‐HTO for varus malalignment performed at a single institution were retrospectively screened for eligibility. Preoperative data were retrieved from medical charts, while incidence and type of MHF were evaluated on postoperative X‐rays. To determine risk factors for MHF, a series of univariate logistic regression were performed using demographic and radiological data as independent variables, using a generalised linear mixed model (GLMM). Variables that demonstrated a significant difference (*p* < 0.1) in univariate analyses were defined as independent variables and were used as covariates in a multivariate analysis with the same dependent variables

**Results:**

A total of 137 knees were included in the study. The incidence MHF fractures was 57% and three distinct types of fractures were identified. The most common fracture type was the two fragment one, with its subtypes “linear” (31%), “distal” (9%) and “proximal” (8%). A “third fragment” MHF was identified in 7% of cases, while “intra‐articular” pattern was observed in 2%. Multivariate analysis showed that increased distance between the end of the osteotomy line up until the medial tibial plateau and decreased length of the osteotomy line were significantly associated with higher probability of MHF: respectively OR (odds ratio) 2.8 [1.20–6.78] (*p* = 0.018) for the former and OR of 0.35 [0.16–0.77] (*p* = 0.009) for the latter.

**Conclusion:**

MHF is a common complication after CW‐HTO, and all the risk factors appear to be related to the osteotomy line: higher MHF incidence was associated with an increased distance from the medial plateau and reduced depth of the cut. Both these parameters are clinically relevant as they are modifiable.

**Level of Evidence:**

Level IV.

**Abbreviations**
ACL: anterior cruciate ligament
BMI: body mass index
CW‐HTO: closing wedge high tibial osteotomy
DFO: distal femur osteotomy
HKA: hip‐knee‐ankle angle
HTO: high tibia osteotomy
MHF: medial hinge fracture
ML: mediolateral
OA: osteoarthritis
OW‐HTO: opening wedge high tibial osteotomy
TKA: total knee arthroplasty

## INTRODUCTION

High tibial osteotomy (HTO) is a well‐established procedure for the treatment of medial knee osteoarthritis in association with varus malalignment [3, 7]. Both lateral closing wedge HTO (CW‐HTO) and medial opening wedge HTO (OW‐HTO) have demonstrated satisfactory clinical and radiological results, delaying the need for total knee replacement (TKR) up to 15 years, with no superiority in clinical outcomes of one technique compared to the other [12, 15]. Recent evidence stated that CW‐HTO has similar clinical and radiological outcomes with respect to OW‐HTO, supporting its use in a modern joint preservation algorithm for varus knee OA [2, 8, 17].

Despite the overall satisfactory clinical results, complications of HTO can be severe and negatively affect the clinical outcome. The complications after CW‐HTO include neurological lesion, particularly common peroneal nerve damage, vascular lesion, loss of correction, non‐union, failure of fixation, superficial or deep infection, and intraoperative fracture [2, 13, 17].

Lateral hinge fractures after OW‐HTO have been extensively studied and have been associated with loss of correction, non‐union, and revision surgery [5], yielding to the identification of risk factors and the development of specific classification systems, such as the Takeuchi classification [18].

In contrast, hinge fractures after CW‐HTO have been less thoroughly investigated, and comparable analyses and evaluations of risk factors are currently lacking [10, 14, 17, 18, 21]. Furthermore, reporting the incidence and classifying the pattern type of these fractures after CW‐HTO can contribute to increasing surgeon awareness on the subject and put the base for a more detailed analysis of biomechanics and risk factors, with the goal of improving surgical planning and technique minimising complication rate.

Given this background, the primary objective of this study was to evaluate the incidence and morphology of medial hinge fractures (MHF) after CW‐HTO and to describe a classification of morphology patterns.

The hypothesis behind the present study was that specific radiological parameters related to the osteotomy line could predispose to MHF in CW‐HTO, therefore the secondary purpose of the study was to identify these factors in order to improve patients' selection process, surgical planning and execution.

## MATERIALS AND METHODS

### Patient selection criteria

This study was conducted using data from the Rizzoli Orthopaedic Institute database. Patients who underwent isolated CW‐HTO for concomitant varus knee malalignment and medial compartment osteoarthritis were screened for inclusion in the analysis. Inclusion criteria were isolated osteotomic procedure, complete clinical records available, complete radiological data (including pre‐operative full‐length weight‐bearing and post‐operative knee radiographs) and age >18 years old at the enrolment in the study. Exclusion criteria included post‐traumatic deformity, revision osteotomy or concomitant procedures (e.g., ACL reconstruction, cartilage procedures), Grade IV OA according to Kellgren–Lawrence and age <18 years old (Figure 3). Demographic data, including sex, age at the time of surgery, and body mass index (BMI), were collected from patients' medical records. Radiological evaluation was conducted using the institutional DICOM viewer software to assess preoperative radiological parameters. Immediate postoperative radiographs were analysed to identify and classify fractures of the medial hinge of the proximal tibia.

### Surgical technique

The planned alignment correction aimed to achieve a postoperative hip‐knee‐ankle (HKA) angle as close as possible to neutral mechanical alignment (HKA 180°–182°).

The CW‐HTO technique has been previously described in detail [2]. A 5–7 cm S‐shaped skin incision is made, extending from the fibular head toward the tibial tubercle. The fascia of the anterior compartment of the tibia is palpated to identify the course of the common peroneal nerve (CPN). After the detachment of the tibialis anterior, the osteotomy site is exposed, and a retractor is placed beneath the tibia to protect the neurovascular bundle and the popliteus muscle.

Under fluoroscopic guidance, two Kirschner wires are inserted from the lateral aspect of the tibia. The first wire is placed parallel to the joint line, while the second is inserted inferiorly at the planned correction angle.

The osteotomy is performed with an oscillating saw along the plane defined by the two K‐wires, and completed with an osteotome. The tibial bone wedge is then removed, followed by a proximal fibular osteotomy. Finally, valgus stress is applied to close the osteotomy gap, and a Krakow staple (Smith & Nephew) is used to stabilise the osteotomy.

### Postoperative management and rehabilitation

Patients were instructed to remain non–weight‐bearing on the operated limb for the first two weeks, followed by toe‐touch weight‐bearing for an additional two weeks, using a knee extension brace during ambulation. In cases of MHF, the non–weight‐bearing period was extended to four weeks.

At one month postoperatively, clinical and radiographic evaluations were performed to assess osteotomy healing, and full weight‐bearing was generally permitted at this stage.

Antithrombotic prophylaxis with enoxaparin was maintained until full weight‐bearing was achieved.

Knee range of motion (ROM) exercises began on postoperative day seven, incorporating both active and passive movements with the assistance of a motorised continuous passive motion device, with full ROM typically regained within six weeks.

Isometric quadriceps strengthening exercises were initiated one week postoperatively, and stationary cycling along with closed kinetic chain exercises were introduced at four weeks.

### Radiographic assessment

**Figure 1:**
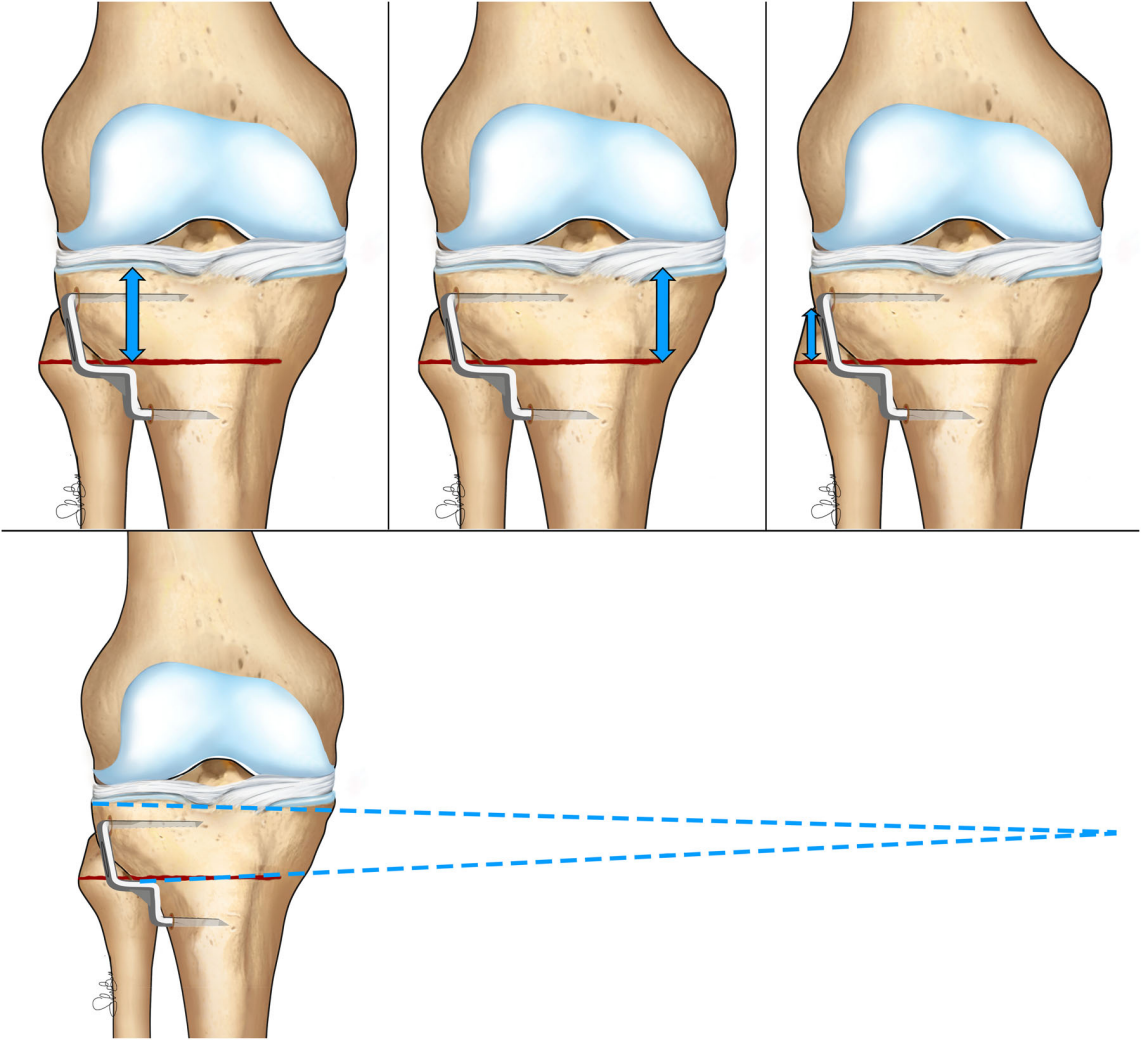
Illustration of the evaluated measurements; upper line, from the left to the right: HTO‐lateral plateau distance, HTO‐medial plateau, HTO‐fibular head distance; lower line: HTO‐tibial plateau angle. HTO, high tibial osteotomy.

**Figure 2:**
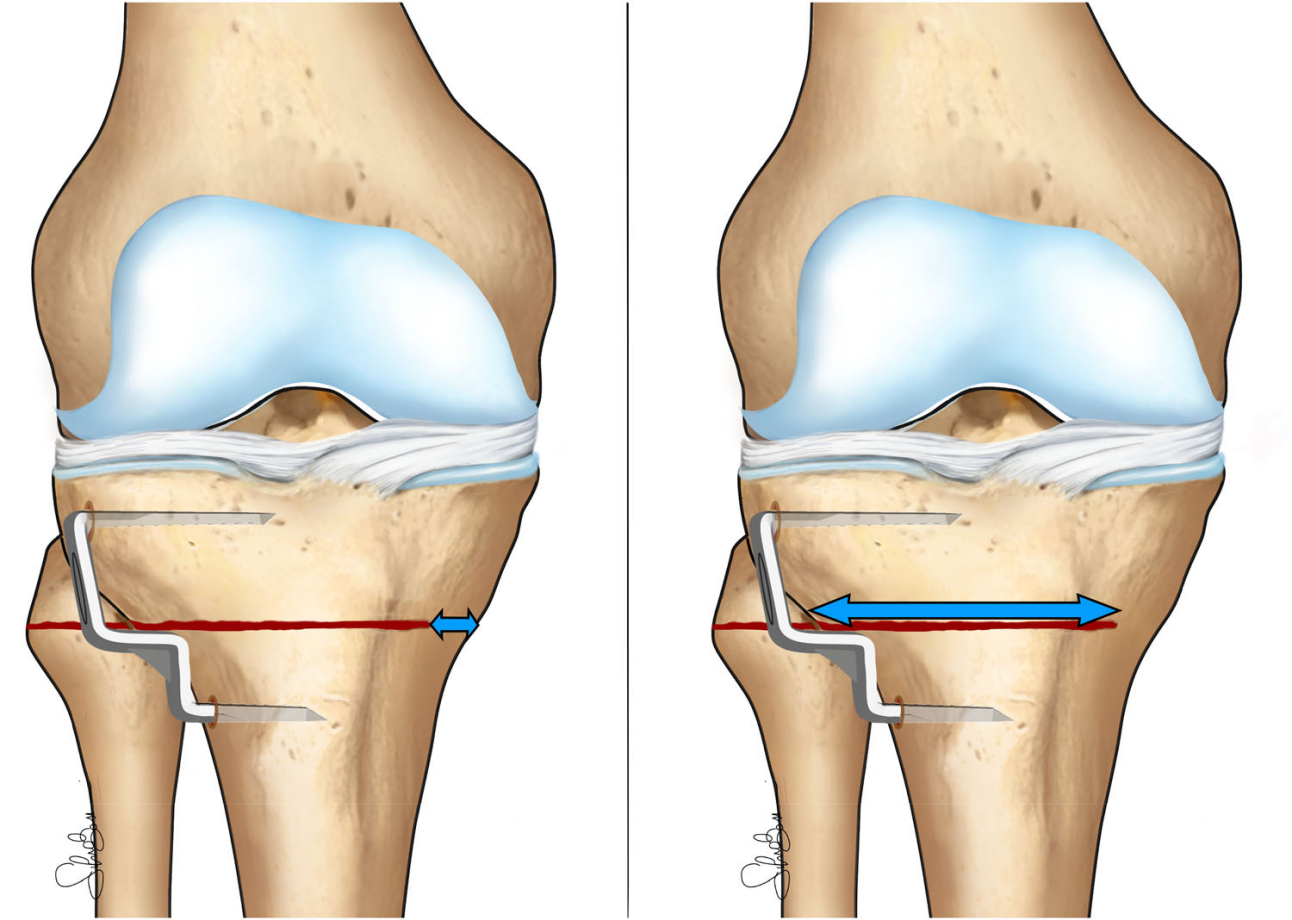
Illustration of the evaluated measurements; from the left to the right: Hinge ML width; HTO ML width. HTO, high tibial osteotomy; ML, mediolateral.

Post‐operative radiological supine frontal X‐rays were evaluated: six different measurements were made for each patient, as listed below (Figures 1 and 2). The measures performed included:

- HTO – lateral plateau distance: the distance between the beginning of the osteotomy line up until the lateral tibial plateau.
- HTO – medial plateau distance: the distance between the end of the osteotomy line up until the medial tibial plateau.
- HTO – fibular head distance: the distance between the top of the fibular head and the level of the osteotomy line.
- HTO – tibial plateau angle: the angle created by the osteotomy line and the tibial plateau.
- HTO ML (medio‐lateral) width: the length of the osteotomy cut.
- Hinge ML (medio‐lateral) width: the length of the medial cortical hinge.

The ratio between the osteotomy cut (HTO ML width) and the entire osteotomy line (HTO ML width + Hinge ML width) was calculated (HTO/Tibial ML width) and noted for statistical analysis.

During this radiological assessment each patient was evaluated for MHF of proximal tibia.

### Statistical Analysis

The statistical analysis was performed using R‐studio (4.3.2, Posit PBC, Wien, Austria). Continuous variables were presented as mean ± standard deviation, while categorical variables were presented as absolute frequencies.

To assess the reproducibility of the proposed fracture classification, interobserver reliability was evaluated between two observers. Each observer independently classified all radiographic cases according to the proposed system, blinded to each other's results. The level of agreement was quantified using Cohen's kappa (κ) statistics, calculated with unweighted categories, as the classification variables were nominal.

Since bilateral cases were included, leading to multiple observations from the same patient, a generalised linear mixed model (GLMM) with a random intercept for each patient was employed to account for intra‐patient correlation, to determine the risk of MHF. The dependent variable was defined as the incidence of MHF while the independent variables were age, sex, BMI, preoperative HKA, planned correction angle, HTO‐lateral plateau distance, HTO‐medial plateau distance, HFO‐fibular head distance, HTO‐tibial plateau angle, Hinge ML‐width, HTO ML width and the ratio between the length of osteotomy cut and the entire tibial plateau (HTO/Tibial ML width).

The variables that demonstrated a significant difference at *p* < 0.1 between the two groups in univariate analyses were defined as independent variables and were used as covariates in a multivariate analysis with the same dependent variables. This threshold was selected to avoid excluding potentially relevant predictors that may become significant in a multivariable context [6].

**Figure 3:**
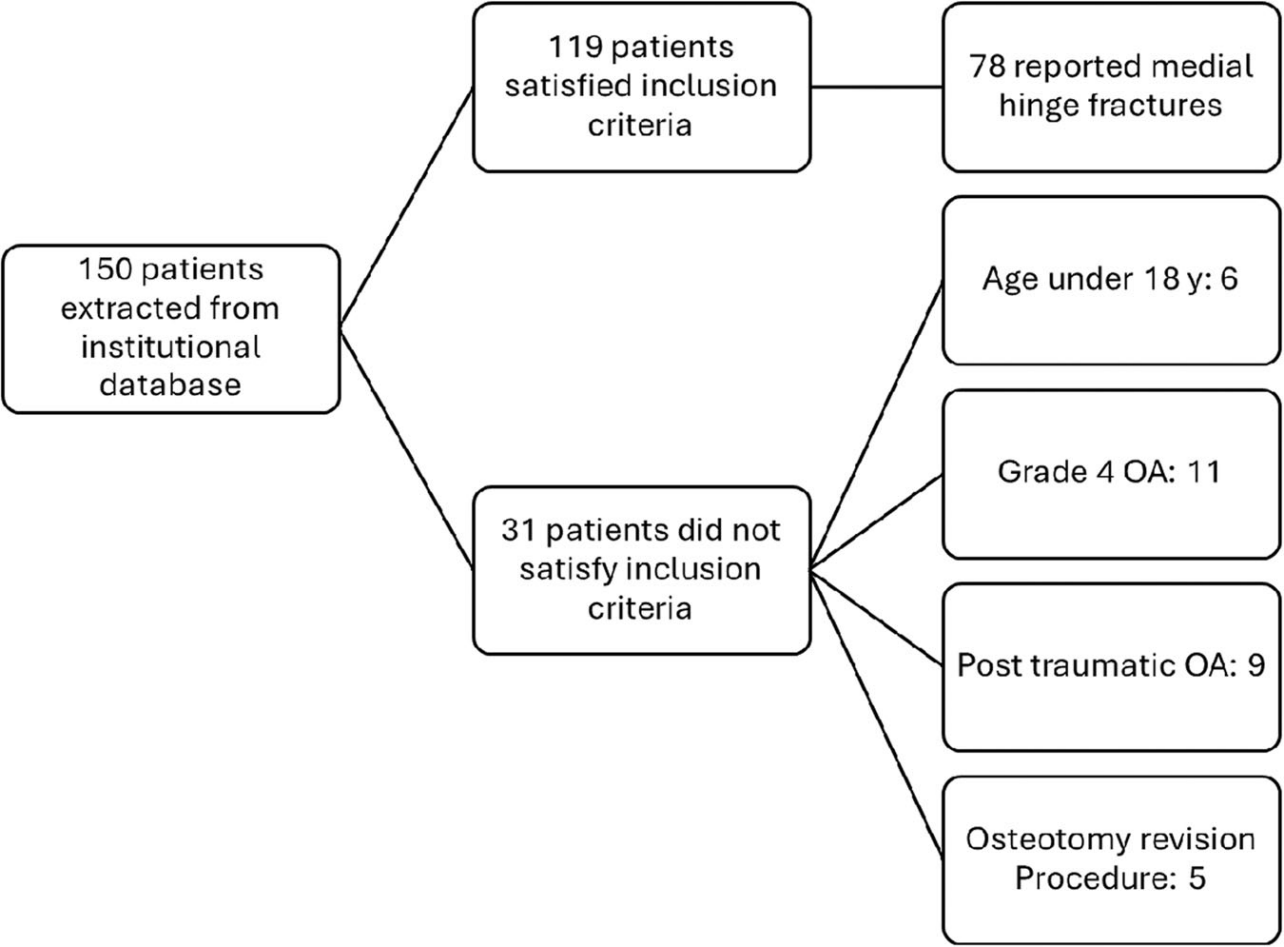
Consolidated Standards of Reporting Trials (CONSORT) diagram. Flowchart of patients included in the present study and reason for their exclusion. OA, osteoarthritis.

An a priori power analysis was conducted to determine adequate sample size in G*Power software (Version 3.1.9.4, 2019, University of Düsseldorf, Germany). An effect size of 0.25 was selected based on estimates derived from contingency tables from Thürig et al. [19] using the chi‐square test. The analysis was conducted with a significance level (*α*) set at 0.05 and a statistical power (1 −* β*) of 0.80. The estimated minimum sample size required to reliably detect this effect was 126 participants (knees) (Figure 3).

## RESULTS

A total of 119 patients (137 knees) were included in the study, with a mean age of 43.7 ± 10.5 years. The majority of the patients were male (115 males, 22 females). The mean BMI of the patients was 25.7 ± 3.5 kg/m². Radiological parameters revealed a mean preoperative hip‐knee‐ankle (HKA) angle of 172.6 ± 3.0° with a mean planned correction angle of 10.9 ± 16.4° (Table 1).

**Table 1:** Demographics and radiological data of the included patients.

	Overall	Hinge fracture group	No fracture group
Patients (knees)	137	78	59
Mean age (y)	43.7 ± 10.5 [41.9–45.5]	44.8 ± 10.2 [42.5–47.1]	42.2 ± 10.8 [39.4–45.0]
Sex (F/M)	22/115	9/69	13/46
Limb (right/left)	65/72	38/40	27/32
BMI (kg/m^2^)	25.7 ± 3.5 [25.0–26.3]	25.5 ± 3.3 [24.7–26.3]	25.9 ± 3.8 [24.7–27.1]
Preop HKA (°)	172.6 ± 3.0 [172.0–173.1]	172.5 ± 3.0 [171.7–173.2]	172.7 ± 3.0 [171.8–173.5]
Planned correction angle (°)	10.9 ± 16.4 [7.9–14.0]	12.2 ± 21.6 [6.8–17.5]	9.3 ± 3.0 [8.5–10.2]
HTO‐lateral plateau distance (mm)	3.0 ± 0.4 [2.9–3.1]	3.0 ± 0.4 [2.9–3.1]	3.0 ± 0.4 [2.9–3.1]
HTO‐medial plateau distance (mm)	2.8 ± 0.5 [2.7–2.9]	2.9 ± 0.5 [2.8–3.0]	2.7 ± 0.5 [2.6–2.8]
HTO‐ fibular head distance (mm)	2.2 ± 0.4 [2.2–2.3]	2.3 ± 0.4 [2.2–2.4]	2.2 ± 0.5 [2.0–2.3]
HTO ‐ tibial plateau angle (°)	4.3 ± 2.9 [3.8–4.8]	4.0 ± 2.8 [3.4–4.7]	4.7 ± 3.0 [4.0–5.5]
Hinge ML width (mm)	2.0 ± 1.3 [1.8–2.2]	2.1 ± 0.8 [2.0–2.3]	1.9 ± 1.7 [1.4–2.3]
HTO ML width (mm)	4.5 ± 0.9 [4.3–4.6]	4.2 ± 0.8 [4.0–4.4]	4.9 ± 0.8 [4.6–5.1]
HTO/tibial ML width	0.7 ± 0.1 [0.7–0.7]	0.7 ± 0.1 [0.6–0.7]	0.7 ± 0.1 [0.7–0.8]

*Note*: Radiological data were presented as mean ± standard deviation [95% CI].

Abbreviations: BMI, Body Mass Index; f, female; HKA, hip‐knee‐ankle angle; HTO, High tibia osteotomy; m, male; ML, mediolateral; y, years.

**Figure 4:**
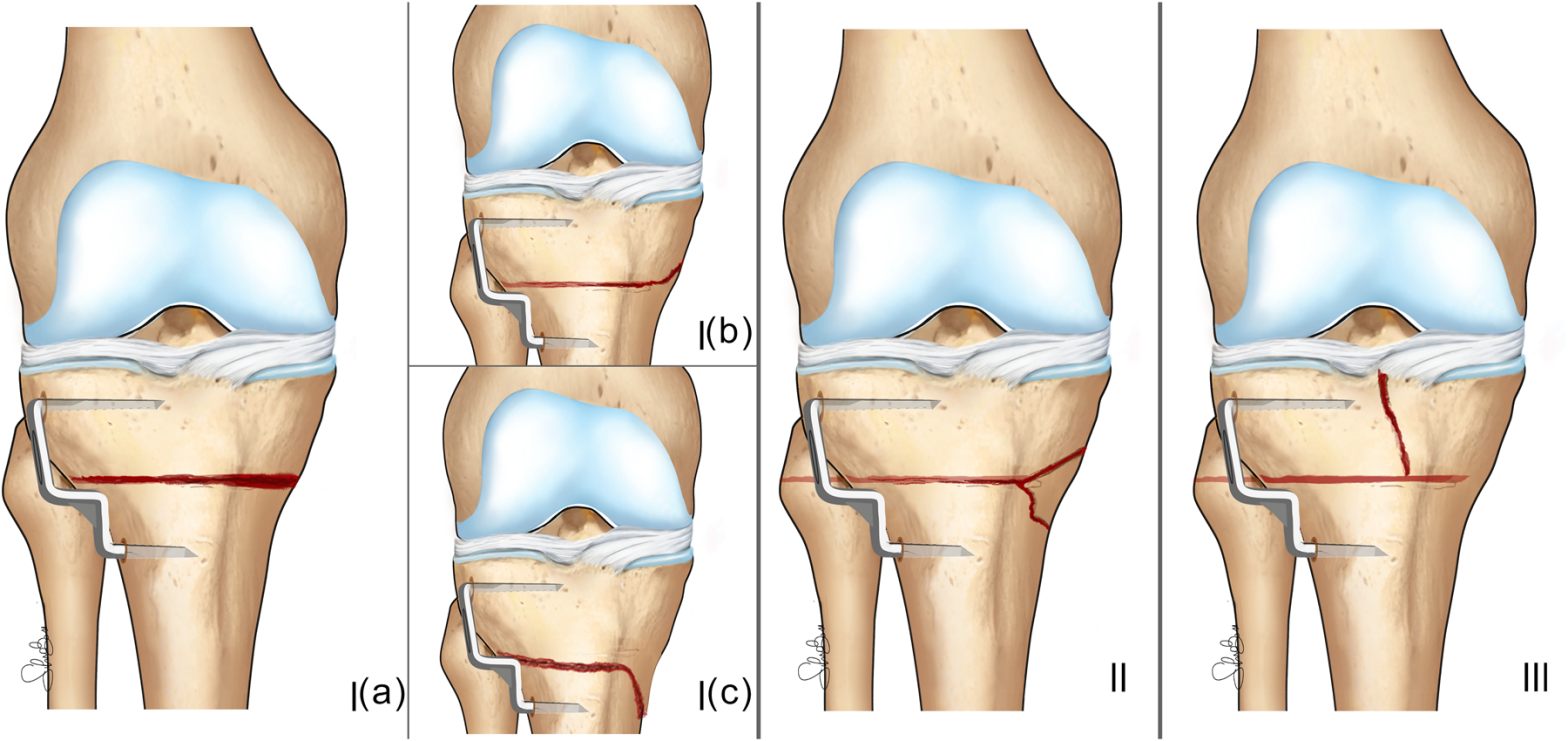
Classification of three distinct types of different medial hinge fracture after Closing‐Wedge high tibial osteotomy, and relative subtypes. Ia: linear fracture; Ib: proximal directed fracture; Ic: distal directed fracture; II: third fragment fracture; III: intra‐articular fracture.

The incidence of medial hinge fractures (MHFs) was 56.9% (78/137). Three distinct fracture types and three subtypes were identified based on the direction and propagation pattern of the fracture line on the medial cortical bone (Figure 4):

- Type 1: two‐fragment fracture. The following three subtypes were identified:

1. Type Ia (“Linear”): the fracture line extends along the osteotomy plane and exits the medial cortex at the same level.
2. Type Ib (“Proximal”): the fracture line extends proximally through the medial cortex, above the level of the osteotomy cut.
3. Type Ic (“Distal”): the fracture line extends distally through the medial cortex, below the level of the osteotomy cut.

- Type II (“Third Fragment”): the fracture line bifurcates before reaching the medial cortex, creating a triangular‐shaped third bone fragment.
- Type III (“Intra‐articular”): the fracture line runs nearly perpendicular to the osteotomy cut and extends toward the articular surface without reaching the medial cortex.

The agreement between the two observers of the proposed fracture classification was almost perfect, with a Cohen's kappa (*κ*) value of 0.94 (95% CI: 0.87–1.00, *p* < 0.001).

The most common fracture type was the type I with 48.1% (66/137) of total population: among the subtypes the most common one was Ia (“linear”) with 31.3% (43/137) of total population followed by Ic (“distal”) 8.7% (12/137) and Ib (“proximal”) 8.0% (11/137). Type II fractures (“third fragment”) was identified in 7.2% (10/137) of patients and type III (“intra‐articular”) pattern directed to the tibial plateau was recorded in 1.4% (2/137) of patients (Table 2).

**Table 2:** Absolute frequencies of the different types of fractures.

Frequencies						
	Type I				Type II	Type III
	Ia – Linear	Ib – Proximal direction	Ic – Distal direction	Overall	Third fragment	Intra‐articular
Overall population (137)	43 (31.3)	11 (8.0)	12 (8.7)	66 (48.1)	10 (7.2)	2 (1.4)
Hinge fracture group (78)	43 (55.1)	11 (14.1)	12 (15.3)	66 (84.6)	10 (12.8)	2 (2.5)

*Note*: Data were presented as (%) with respect to the total *n*. of fracture or *n*. of events/total cases.

According to generalised linear mixed model analysis, HTO‐medial plateau distance, HTO ML width, and HTO/tibial ML Width were used as independent variables for inclusion in the multivariate analysis with hinge fracture as the dependent variable (Table 3). Multiple generalised linear mixed model analysis indicated that increased HTO‐Medial Plateau Distance and decreased HTO ML Width and were significantly associated with higher occurrence of hinge fractures (Table 3).

**Table 3:** Univariate (left column) and multiple (right column) generalised linear mixed model analysis in relation to medial hinge fracture.

	Univariate analysis			Multivariate analysis		
	Odds ratio	CI 95%	*p* value	Odds ratio	CI 95%	*p* value
Mean age (y)	1.03	[0.99–1.07]	0.215	‐		
Sex (F/M)	0.64	[0.37–1.12]	0.123	‐		
Limb (right/left)	1.07	[0.72–1.59]	0.752	‐		
BMI (kg/m^2^)	0.99	[0.94–1.03]	0.496	‐		
Preop HKA (°)	0.97	[0.86–1.11]	0.694	‐		
Planned correction angle (°)	1.03	[0.93–1.15]	0.574			
HTO‐lateral plateau distance (mm)	1.11	[0.40–3.08]	0.835	‐		
HTO‐medial plateau distance (mm)	2.59	[1.11–6.02]	0.028	2.85	[1.20–6.78]	0.018
HTO‐fibular head distance (mm)	2.04	[0.79–5.27]	0.143	‐		
HTO‐tibial plateau angle (°)	0.91	[0.78–1.05]	0.175	‐		
Hinge ML width (mm)	1.19	[0.86–1.65]	0.294	‐		
HTO ML width (mm)	0.28	[0.14–0.59]	<0.001	0.35	[0.16–0.77]	0.009
HTO/tibial ML width	0.00	[0.00–0.14]	0.002	0.17	[0.00–11.59]	0.410

*Note*: Data were presented as odds ratio. Factors were included in the multivariate analysis if with *p* < 0.1 at the univariate analysis.

Abbreviations: HTO, high tibial osteotomy; ML, mediolateral.

## DISCUSSION

The key findings of this study are that medial hinge fractures (MHF) occur in more than half of the patients undergoing closing wedge high tibial osteotomy (CW‐HTO). Additionally, this research has for the first time identified and described different patterns of MHF, providing a classification system for these fractures in the context of CW‐HTO.

A comprehensive analysis was conducted on both patient‐related and surgical‐related risk factors for MHF. The results revealed that an increased HTO‐medial plateau distance (i.e., the height of the osteotomy cut relative to the joint line) and a reduced HTO‐ML width of the cut (i.e., the depth of the cut) were significantly associated with higher MHF in the multivariate analysis.

These two modifiable surgical parameters are clinically significant due to their direct influence on surgical outcomes. The findings of this study provide valuable insights for surgeons, enabling a critical evaluation of their surgical planning. By avoiding an excessive distal cut and ensuring the preservation of an adequate hinge before osteotomy closure, surgeons can potentially reduce the incidence of hinge fractures.

This study present a novel classification of MHF after CW‐HTO; this complication has been seldom explored in literature, as most studies on CW‐HTO have not documented the occurrence of this fracture [9]. To the best of our knowledge, only one previous study has reported on medial hinge fractures after CW‐HTO, reporting an incidence of 82%, with 36 out of 44 patients affected [14]. This rate is surprisingly high when compared to medial OW‐HTO, implying that hinge fractures are often regarded as an almost inevitable consequence of the surgery rather than a significant complication. Nonetheless, due to the absence of a formal classification system, hinge fractures have not been systematically investigated, even though different fracture patterns may lead to different clinical outcomes.

Research investigating the immediate postoperative incidence of lateral hinge fractures after medial opening wedge HTO has reported a variable incidence ranging between 3% and 30%, with most studies reporting a value around 20% [12].

In the present study, the most common type of fracture was the “two fragment” type, and in particular the subtype with a linear extension of the osteotomy cut, occurring in 31% of patients. These findings are consistent with the Takeuchi classification for lateral hinge fractures after OW‐HTO, which also identified a predominance of linear fractures in OW‐HTO [11]. Similar results were also reported by Winkler et al. in a retrospective study evaluating the incidence of contralateral hinge fractures after OW‐DFO [20].

The different MHF patterns may also have practical implications for rehabilitation and the risk of mechanical complications. In two‐fragment fractures, the wide bone contact and preservation of soft‐tissue continuity may favour early consolidation and provide intrinsic stability, allowing standard non‐weight‐bearing for 4 weeks and gradual loading after radiographic control, with a low risk of malalignment or non‐union, and complications may arise only in the presence of hinge disruption or displacement [16].

An interesting fracture pattern, not previously described in any osteotomy hinge fracture classification system, was the “third fragment” pattern, observed in 7% of cases. In this pattern, two separate fracture lines arise from the initial osteotomy cut: one extending proximally and the other distally, creating a “third fragment” the medial cortical hinge. Those fractures types may be mechanically less stable because the periosteum is disrupted at two points, similar to wedge‐type fractures in the AO classification. In such cases, delayed weight‐bearing and the use of angular stable fixation or additional compression screws might be advisable to reduce the risk of malalignment or delayed union.

The intra‐articular type of fracture was the rarest, observed in only 1.5% (2/137) of CW‐HTO cases. Notably, both patients with this fracture type had undergone prior ACL reconstruction. The fracture line was atypically lateral, following the trajectory of the previous ACL tibial tunnel.

Intra‐articular fractures (Type III) represent the most critical scenario, direct screw fixation and extended protection from weight‐bearing could be recommended in those cases. Although clinical follow‐up data were not available to confirm these hypotheses, these considerations highlight the potential usefulness of the proposed classification for guiding postoperative management and warrant future outcome‐based studies especially since CW‐HTO procedures are still often performed with various fixation devices, such as the Krackow staple, which does not provide angular stability [1].

These findings underline that MHF is a multifactorial complication, highlighting the importance of individualised patient evaluation and meticulous preoperative planning to mitigate the risk of hinge fractures.

The present study has several limitations. Firstly, its retrospective observational design: HTO, and CW‐HTO in particular, is a surgical procedure that is not performed frequently; therefore, the retrospective nature of the data was necessary to reach a sufficient statistical sample. Additionally, the radiographic classification was performed on immediate postoperative knee radiographs. Although radiographs are routinely performed in the postoperative period and a radiographic classification could ensure a more widespread use, it is well acknowledged that many studies recommend CT scans as the gold standard for identifying MHF after HTO, even though CT scans are not routinely performed [12], mainly due to resource limitations, as well as increased radiological risk for the patient. Therefore, it is possible that the incidence of MHF may have been underestimated.

Moreover, this study only presented a morphological classification of postoperative MHF without providing clinical data or outcomes. However, this is the first study to describe MHF after CW‐HTO, and follow‐up studies are necessary to evaluate whether the presence of MHF and the different fracture patterns could be related to inferior clinical outcomes, revision surgery, under‐ or over‐correction.

Another limitation is the relatively small number of patients included in some specific subgroups of fracture patterns. Nevertheless, this issue is common when dealing with fracture classifications. For instance, in the original Takeuchi study, only five patients had a type 2 fracture and two patients had a type 3 fracture [18]. Lastly, the percentage of female patients in the present study was only 16%, which further increases the gender‐data gap in orthopaedic literature [4]. However, both genders were eligible for inclusion in this study, and the increased incidence of tibia vara in male patients is recognised in the literature.

## CONCLUSION

MHF is a common complication following CW‐HTO, with three distinct morphological patterns and relative subtypes. All identified risk factors appear to be associated with the characteristics of the osteotomy cut whether than other demographic or radiological factors. Specifically, a greater distance of the end of the osteotomy cut from the medial tibial plateau and a shorter osteotomy cut are significantly associated with higher occurrence of MHF. Further studies are necessary to investigate the prognostic value of the proposed classification in relation to clinical outcomes, such as survivorship from failure, pseudoarthrosis and loss of correction. The findings of this study provide useful insights for surgeons, giving basic but valuable indications regarding surgical planning and providing a new classification of a common complication.

## AUTHOR CONTRIBUTIONS

Gian Andrea Lucidi conceptualise the idea of the study. Giovanni Balboni and Luca Solaro collected data. Gian Andrea Lucidi, Giovanni Balboni, and Luca Solaro wrote the main manuscript text. Stefano di Paolo and Margherita Bonaiuti were in charge of data evaluation and statistical analysis. Stefano Zaffagnini reviewed the article and supported the team during all phases of work.

## CONFLICT OF INTEREST STATEMENT

The authors declare no conflict of interest.

## ETHICS STATEMENT

This study has been approved by the Local Ethical Committee of Rizzoli Orthopaedic Institute, Bologna, Italy (Protocol No. 887/2022/Oss/IOR). All patients signed informed consent statement in order to participate in this study.

## Supporting information

Appendix ‐ fractures.

Appendix ‐Measures.

## Data Availability

The data that support the findings of this study are available from the corresponding author upon reasonable request.
