# Supplementary material for: Medial hinge fracture after closing wedge high tibial osteotomy: Proposing a new classification and risk factor analysis of a neglected complication
Source: Knee Surg Sports Traumatol Arthrosc. 2025 Dec 8;34(3):1014–23. doi: 10.1002/ksa.70224 (PMC12948340; doi:10.1002/ksa.70224)
Supplement: Supplementary file 1 — Appendix ‐ fractures. [file KSA-34-1014-s001.zip › legend figures appendix.docx]

Fig 1 HTO - lateral plateau distance: the distance between the beginning of the osteotomy line up until the lateral tibial plateau

Fig 2 HTO – medial plateau distance: the distance between the end of the osteotomy line up until the medial tibial plateau

Fig 3 HTO – fibular head distance: the distance between the top of the fibular head and the level of the osteotomy line

Fig 4 Hinge ML (medio-lateral) width: the length of the medial cortical hinge

Fig 5 HTO ML (medio-lateral) width: the length of the osteotomy cut

Fig 6 HTO – tibial plateau angle: the angle created by the osteotomy line and the tibial plateau

Fig 7 Fracture type Ia: two-fragments linear pattern

Fig 8 Fracture type Ib: two-fragments proximal direction pattern

Fig 9 Fracture type Ic: two-fragments distal direction pattern

Fig 10 Fracture type II: third fragment pattern

Fig 11 Fracture type III: intra-articular pattern
