# Supplementary figures and images for: Medial hinge fracture after closing wedge high tibial osteotomy: Proposing a new classification and risk factor analysis of a neglected complication
Source: Knee Surg Sports Traumatol Arthrosc. 2025 Dec 8;34(3):1014–23. doi: 10.1002/ksa.70224 (PMC12948340; doi:10.1002/ksa.70224)

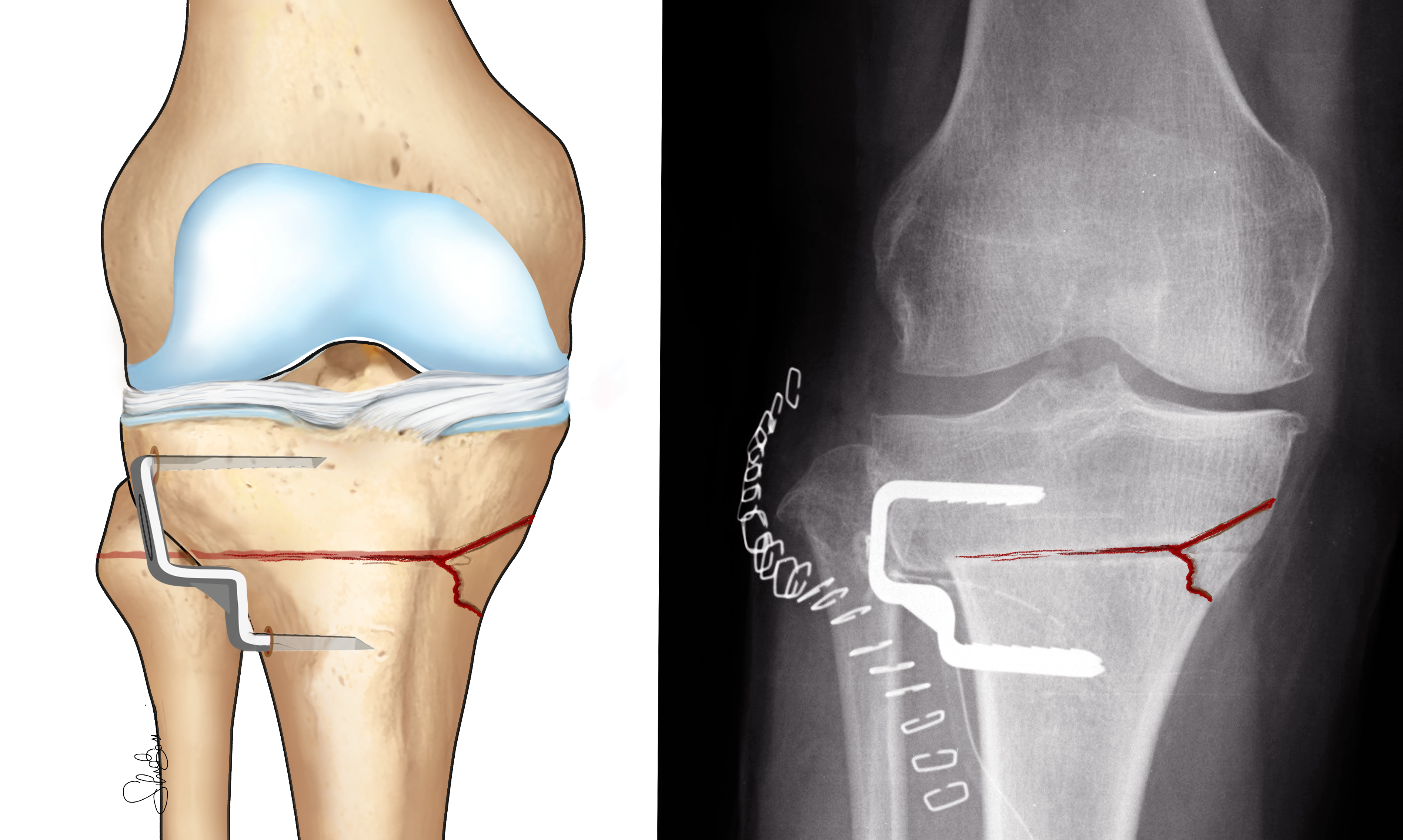

Supplement: Supplementary file 1 — Appendix ‐ fractures. [file KSA-34-1014-s001.zip › fig 10.tif]

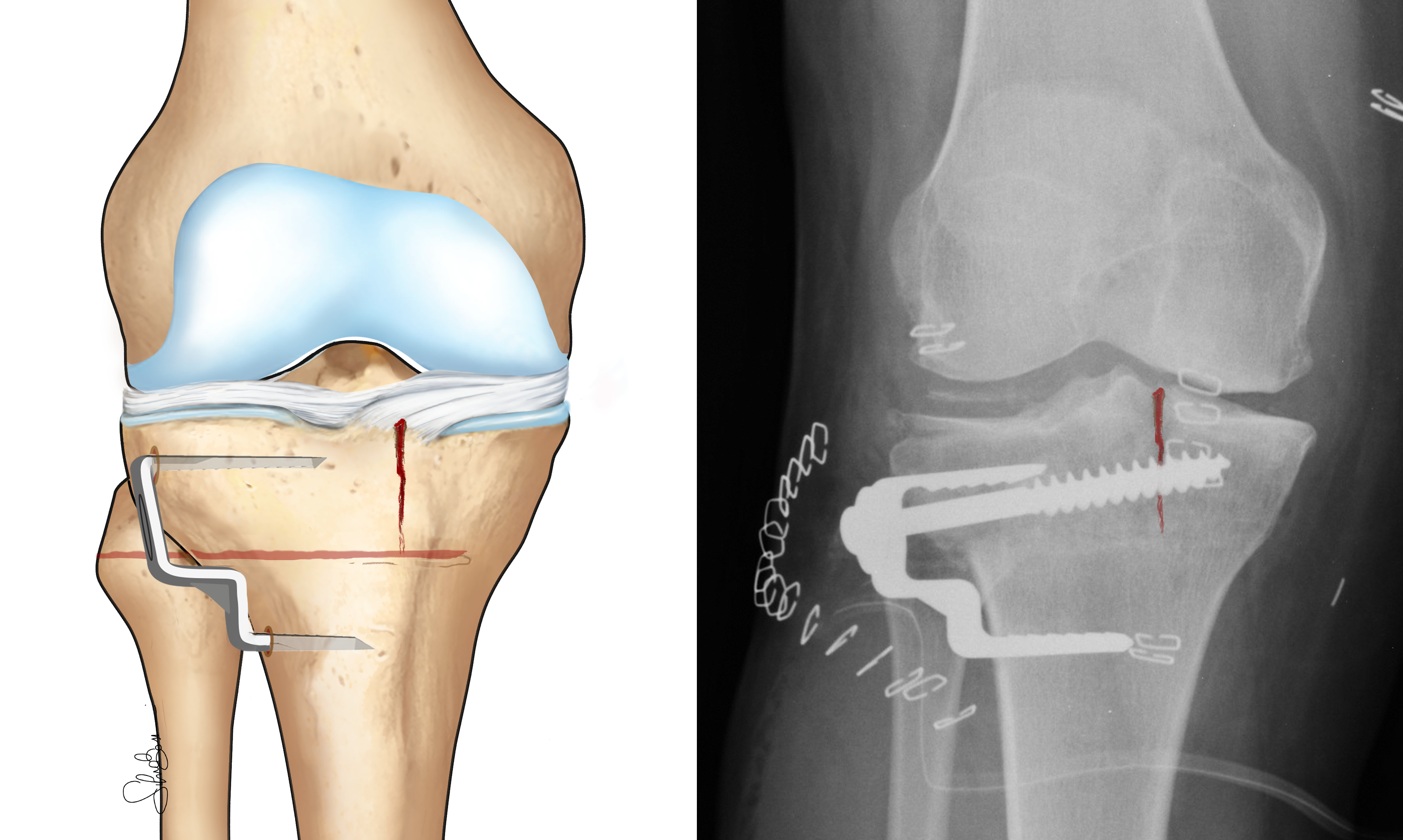

Supplement: Supplementary file 1 — Appendix ‐ fractures. [file KSA-34-1014-s001.zip › fig 11.tif]

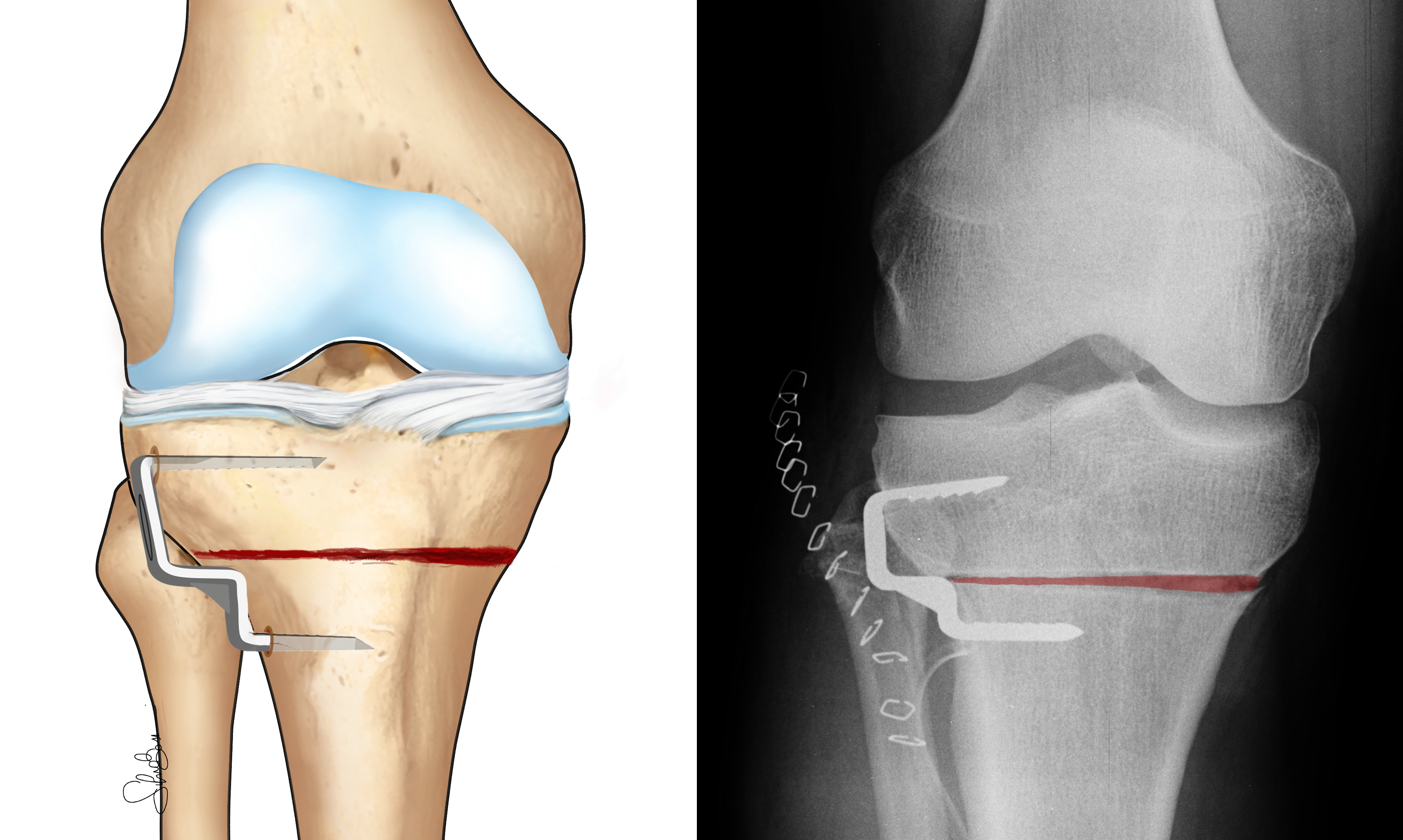

Supplement: Supplementary file 1 — Appendix ‐ fractures. [file KSA-34-1014-s001.zip › fig 7.tif]

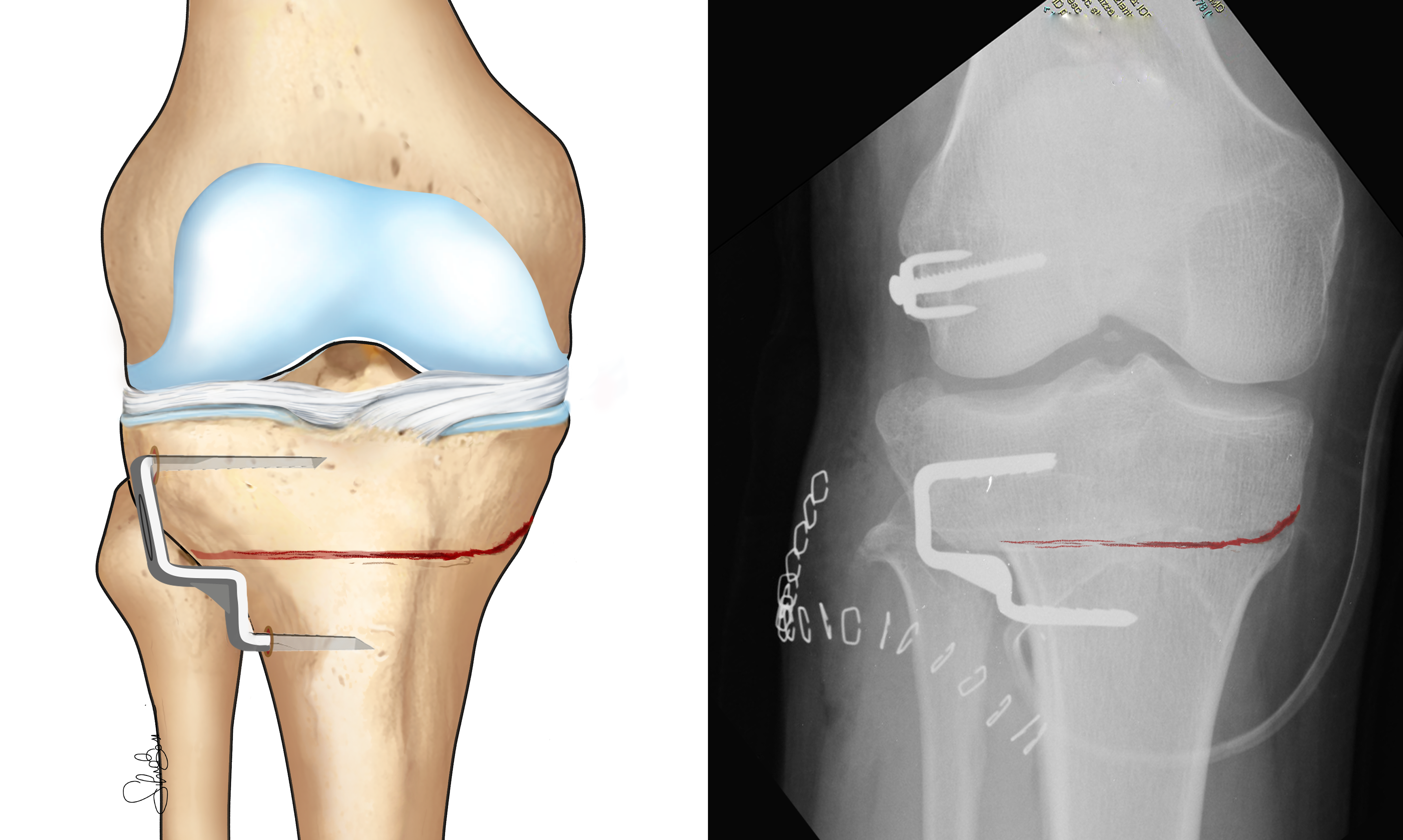

Supplement: Supplementary file 1 — Appendix ‐ fractures. [file KSA-34-1014-s001.zip › fig 8.tif]

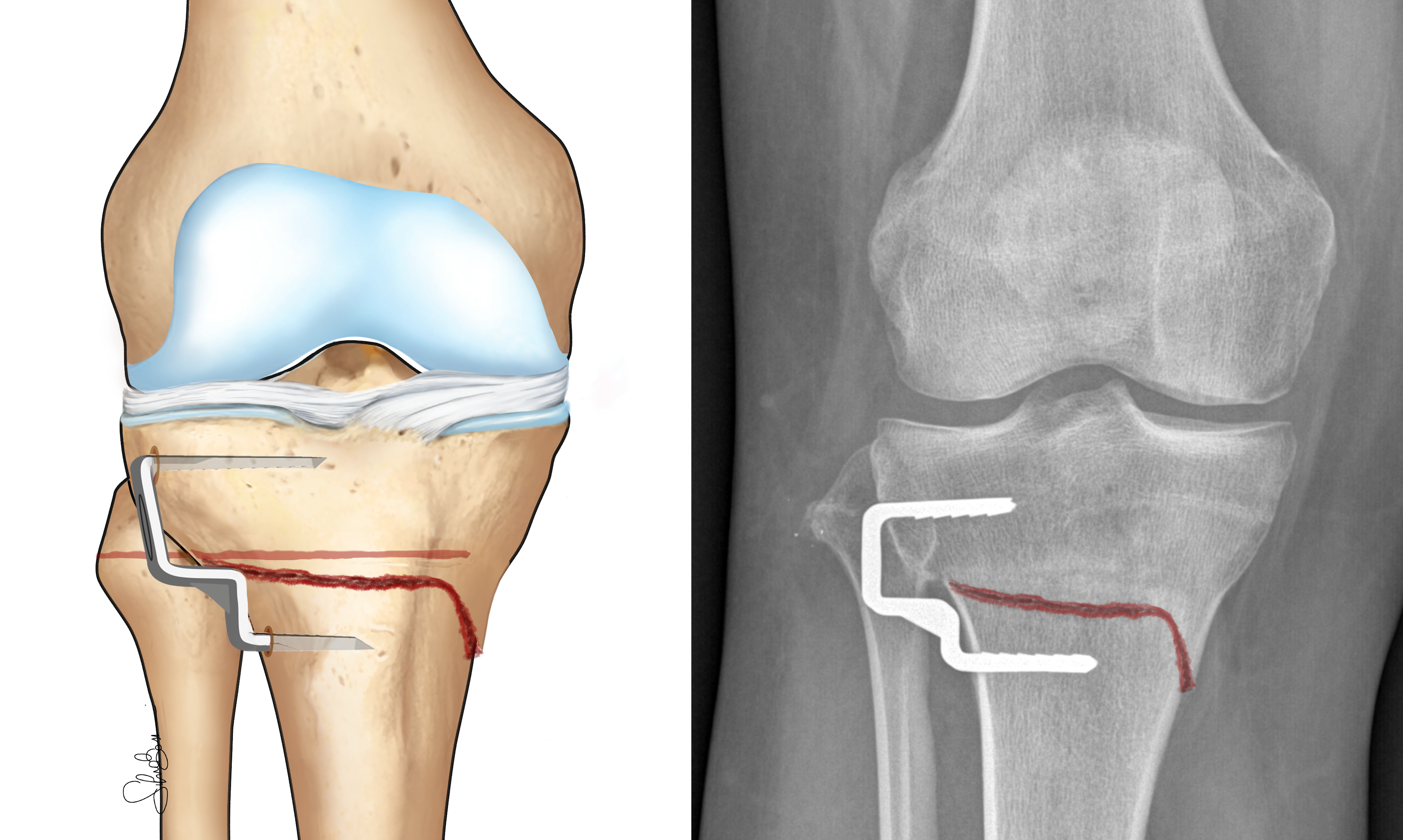

Supplement: Supplementary file 1 — Appendix ‐ fractures. [file KSA-34-1014-s001.zip › fig 9.tif]

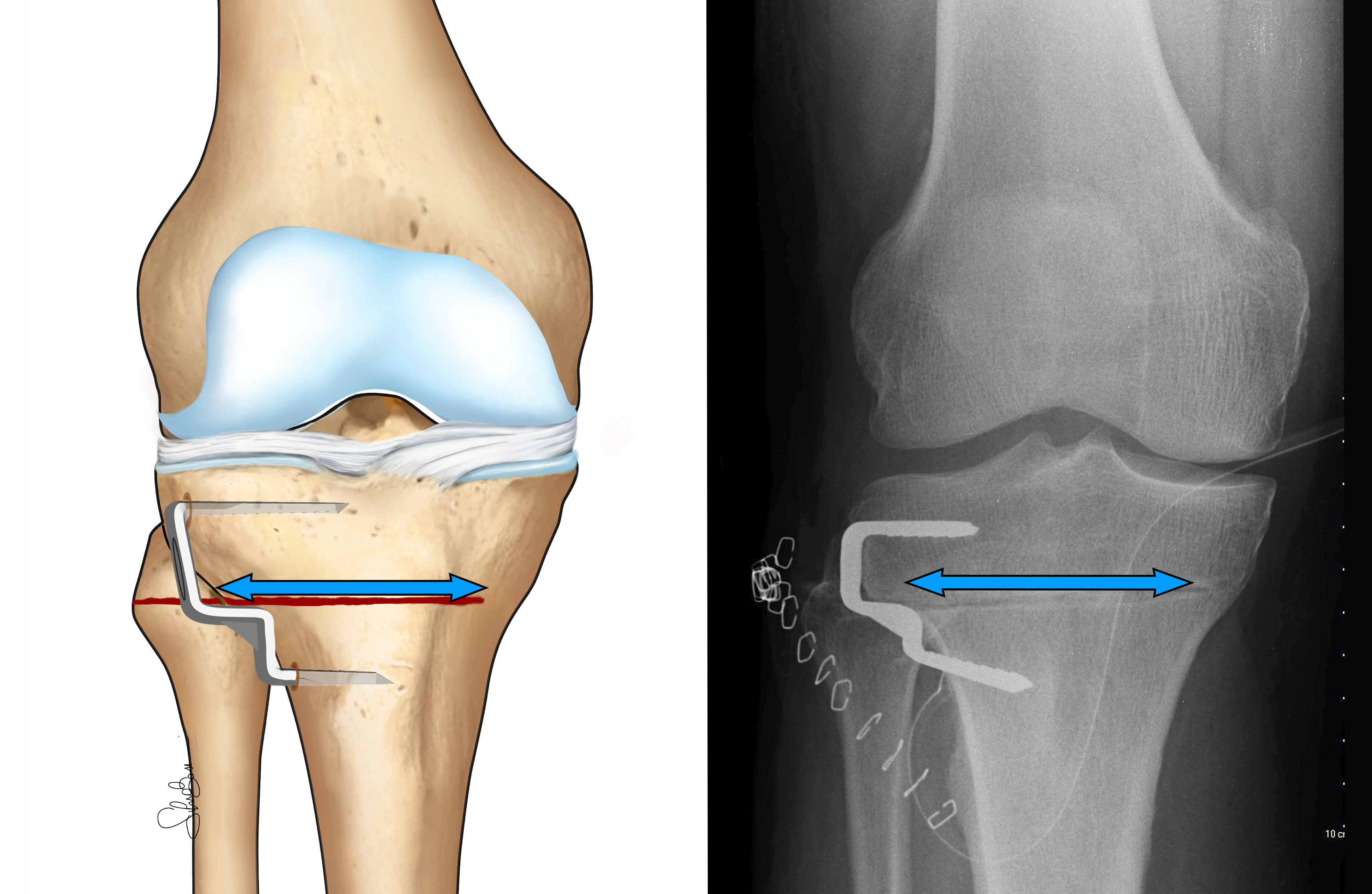

Supplement: Supplementary file 1 — Appendix ‐ fractures. [file KSA-34-1014-s001.zip › fig 5.tif]

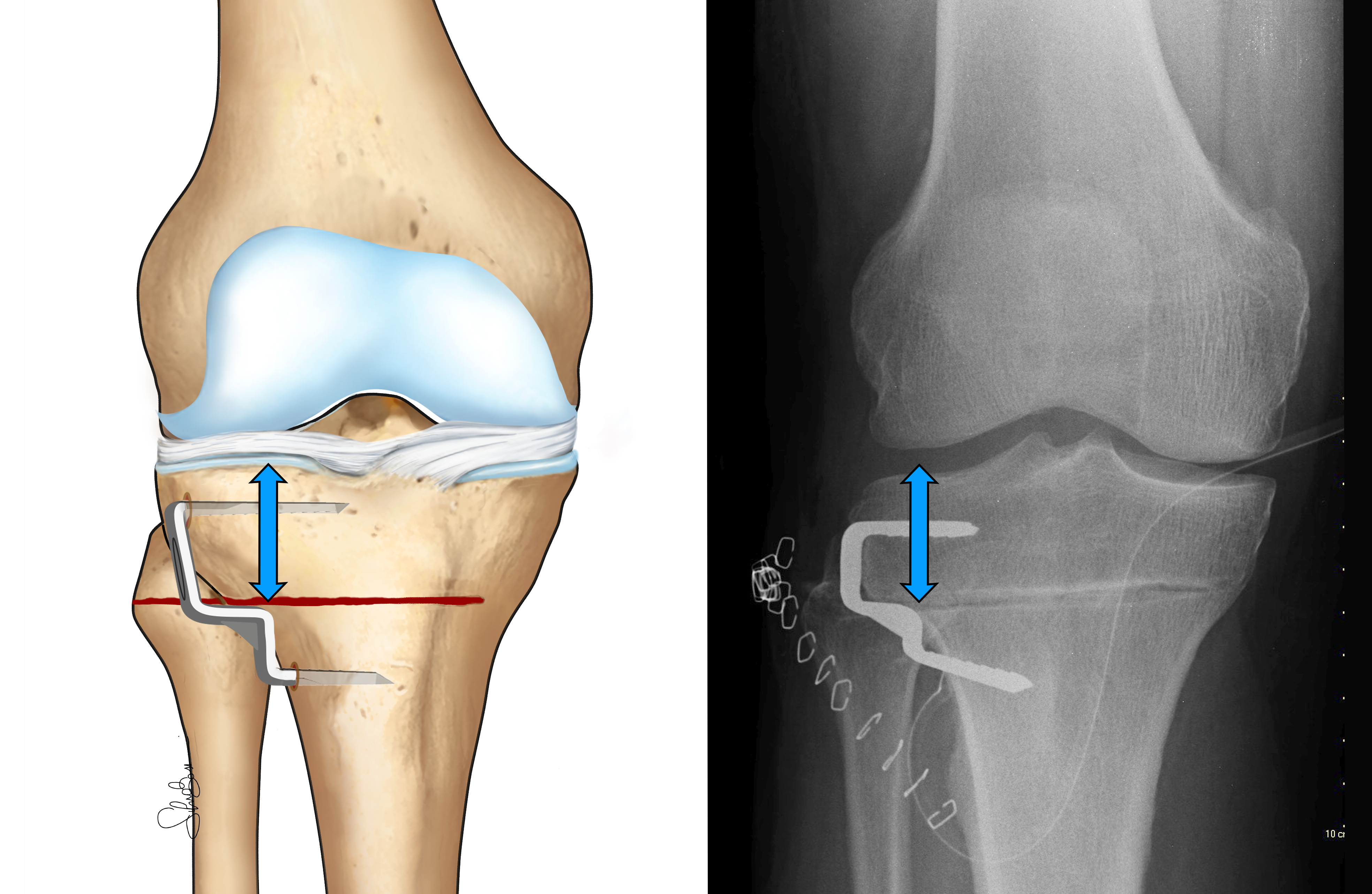

Supplement: Supplementary file 2 — Appendix ‐Measures. [file KSA-34-1014-s002.zip › fig 1.tif]

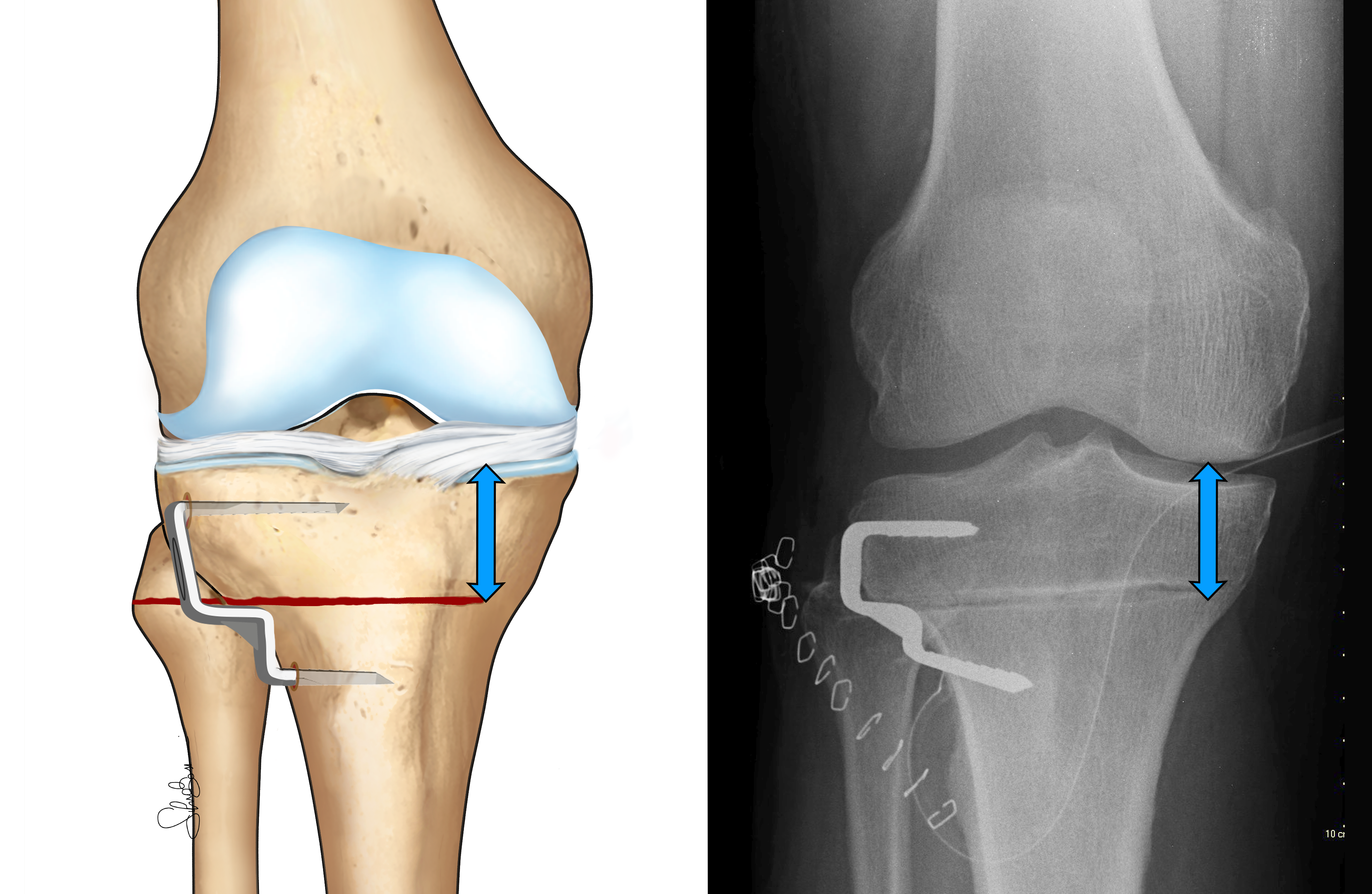

Supplement: Supplementary file 2 — Appendix ‐Measures. [file KSA-34-1014-s002.zip › fig 2.tif]

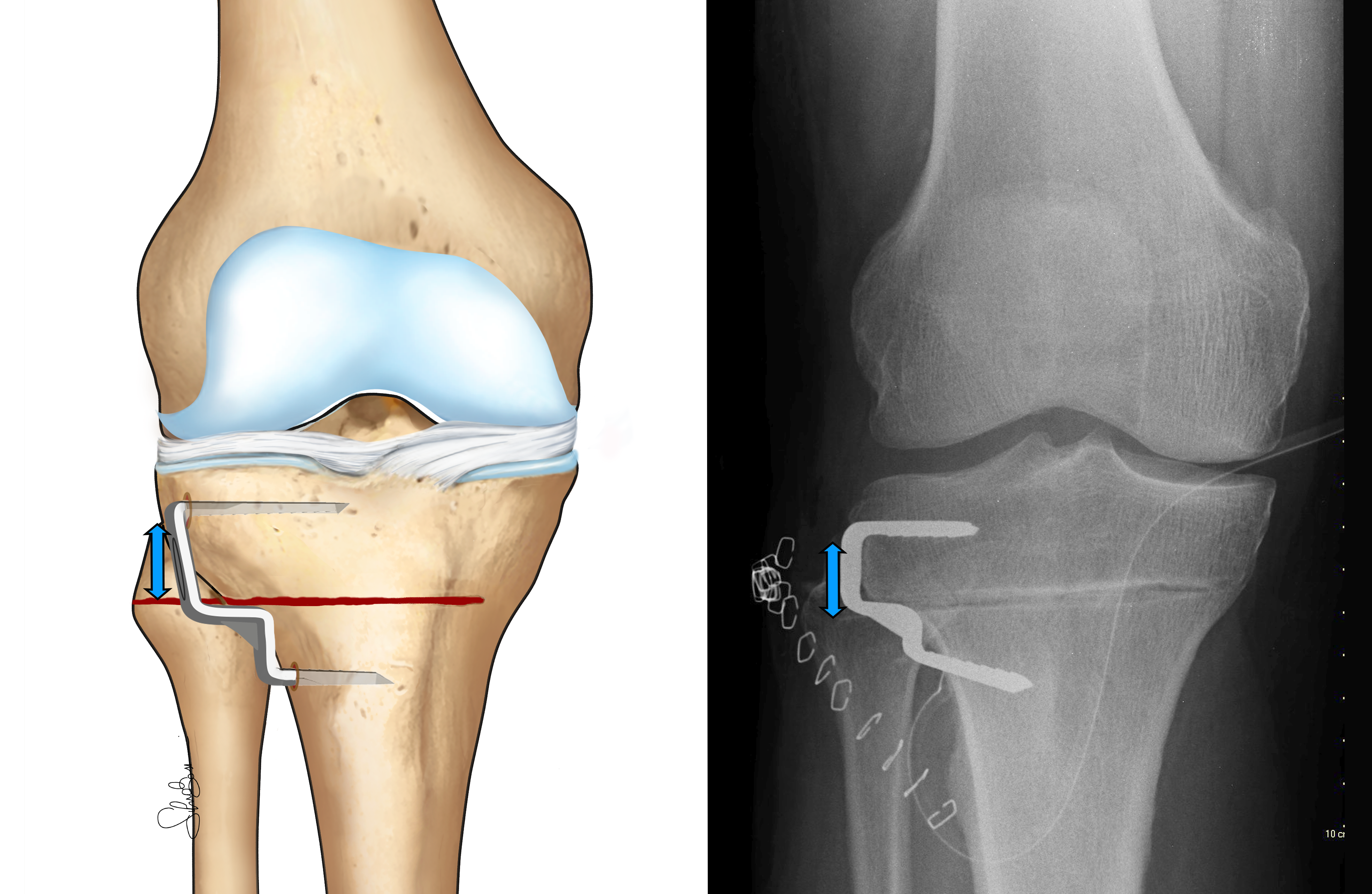

Supplement: Supplementary file 2 — Appendix ‐Measures. [file KSA-34-1014-s002.zip › fig 3.tif]

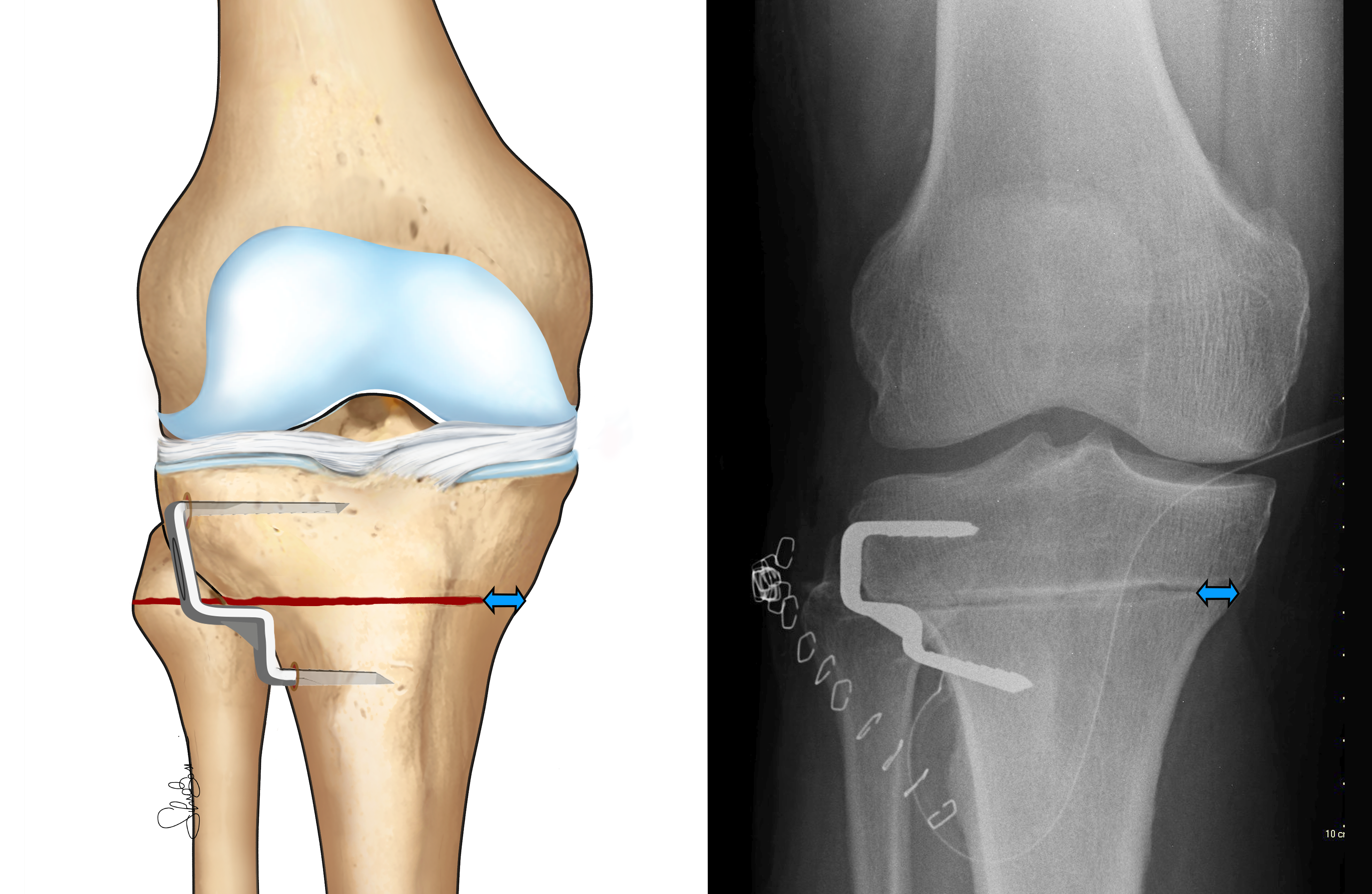

Supplement: Supplementary file 2 — Appendix ‐Measures. [file KSA-34-1014-s002.zip › fig 4.tif]
